# Bilateral Suspected Tuberculous Empyema Thoracis

**Published:** 2012-06-01

**Authors:** Yousuf Aziz Khan

**Affiliations:** Department of Pediatric Surgery, National Institute of Child Health Karachi, Pakistan.

**Keywords:** Empyema thoracis, Tuberculosis, Tube thoracostomy

## Abstract

Empyema thoracis is a well known complication following para-pneumonic effusions in paediatric age group. Usually it is unilateral but rarely could be bilateral. Herein we report a case of bilateral tuberculous empyema thoracis in a 12 years old, unvaccinated girl with a positive history of contact with tuberculosis. She was managed conservatively with tube thoracostomies and anti-tuberculous drugs. Emphasis is on the conservative approach and patience in management of patients with bilateral empyema thoracis.

## INTRODUCTION

Empyema thoracis (ET) may occur following trauma, thoracic surgery or iatrogenic esophageal perforation etc, but in children mostly it follows complicated para-pneumonic effusions [1]. The primary empyema thoracis occurs without underlying infection [2]. In a developing country such as ours, tuberculosis is one of the commonest causes of empyema thoracis. Usually it is unilateral, mostly involving right side. Rarely it involves both the pleural spaces, the bilateral empyema thoracis (BET) [3]. Here in a case of bilateral tuberculous empyema thoracis in a young girl is reported that was managed conservatively.

## CASE REPORT

A 12-year-old girl presented with gradual onset of continuous, low to high grade fever, and cough which was initially non-productive but later productive of yellow sputum over 20 days. She had anorexia and lost weight. She developed respiratory distress which gradually worsened. Family history was significant for tuberculosis in grandmother who lived with her. She was unvaccinated. Initially they took treatment from a family physician but later referred to other facility where she was admitted and worked up as no improvement was noted. Her x-ray chest showed bilateral pleural effusions (Fig.1) and ultrasound of the chest revealed large effusions on both sides with thick internal echoes. At thoracocentesis, pus was aspirated from both the sides. She was started on anti-tuberculous treatment (Inj. streptomycin, isoniazid, rifampicin and pyrizinamide). After the thoracocentesis, her condition worsened and x-ray chest revealed left pneumo-thorax, (Fig.2) and she was referred to our centre.

**Figure:**
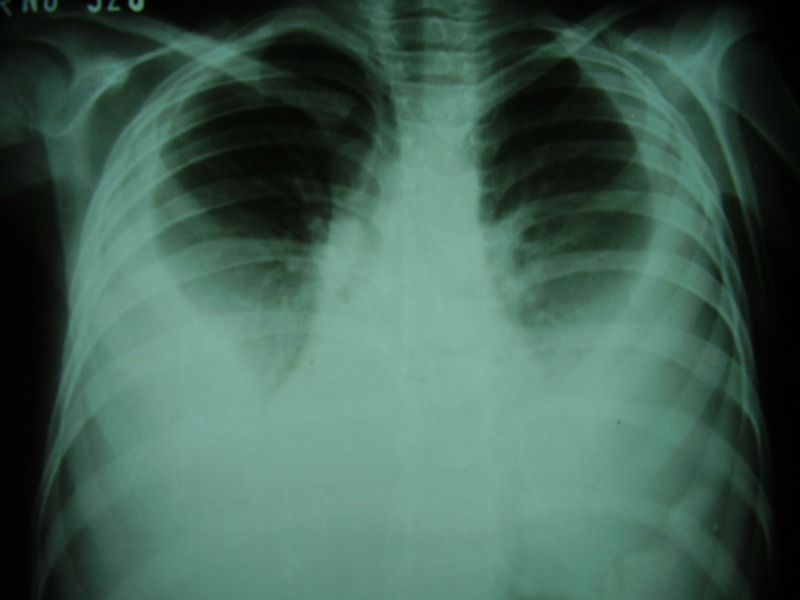
Figure 1: Bilateral pleural effusions.

**Figure:**
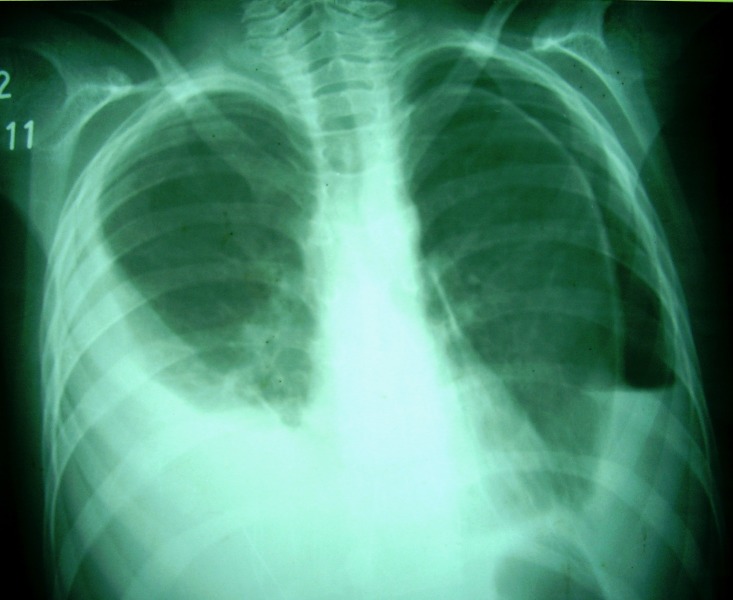
Figure 2: Bilateral pleural effusions with pneumothorax on left side.

At arrival, examination revealed a thin, emaciated, 21 kgs, tachypneic girl, with a respiratory rate of 52/min and heart rate of 118/min. BCG scar was not found. Chest movements were equal but air entry was reduced on both sides. Bilateral tube thoracostomies were offered. About 250 cc thin yellow pus was drained initially from the right side and 20 cc thick yellow pus drained from the left side along with air-leak. Post intubation x-ray chest showed partial lung expansion on the right side (Fig.3).

**Figure:**
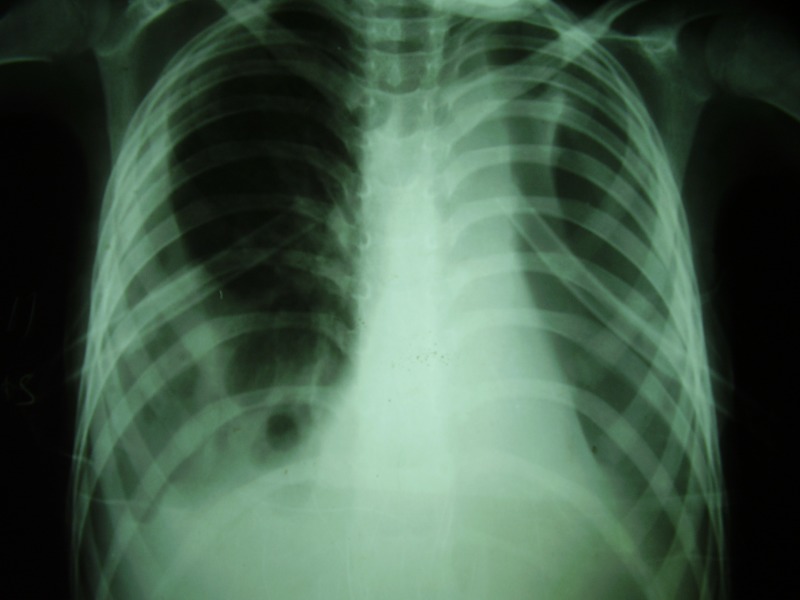
Figure 3: Post intubation chest X-ray.

Laboratory investigations showed Hemoglobin of 9 gm/dl and ESR of 40 mm/1st hour. She was started on ceftazidime and amikacin injectables, empirically along with anti-tuberculous drugs and supportive treatment. The initial pleural fluid examination revealed numerous WBCs, proteins 5.7 gm%, and gram negative rods. Pseudomonas aeruginosa was isolated from the pus and no AFB was seen on Ziehl Nelson (ZN) staining. Antibiotics were changed to tazobactam (according to culture report) and anti-tuberculous drugs continued.

Her condition worsened despite optimal medical treatment. Respiratory distress increased together with persistent air leak and oxygen desaturation. She was shifted to intensive care unit and x-ray chest was repeated which showed bilaterally well expanded lungs with pneumonic patches. Both chest tubes were in place that drained pus though she required re-adjustments multiple times. She was also given nutritional supplementation. The pus culture were repeated that grew Morganilla morgani sensitive to tazobactam.

Gradually her condition settled. Respiratory distress improved and fever subsided. Repeat x-ray chest showed bilaterally well expanded lungs except for a cavitatory lesion at the right lower zone. Ultrasound (US) chest showed collection with internal echoes measuring 5.4 cm × 4.5 cm, and 10 cc pus was aspirated under US guidance and sent for culture. Proteus vulgaris was isolated with same sensitivity pattern. At 42nd and 48th day of intubation, left and right sided chest tubes were removed respectively after the x-rays when the patient was asymptomatic (Fig. 4). She was sent home on anti-tuberculous treatment and vitamin supplements after a total hospital stay of 66 days. On last telephonic conversation with family the patient was reported as thriving well, gained weight and was asymptomatic.

**Figure:**
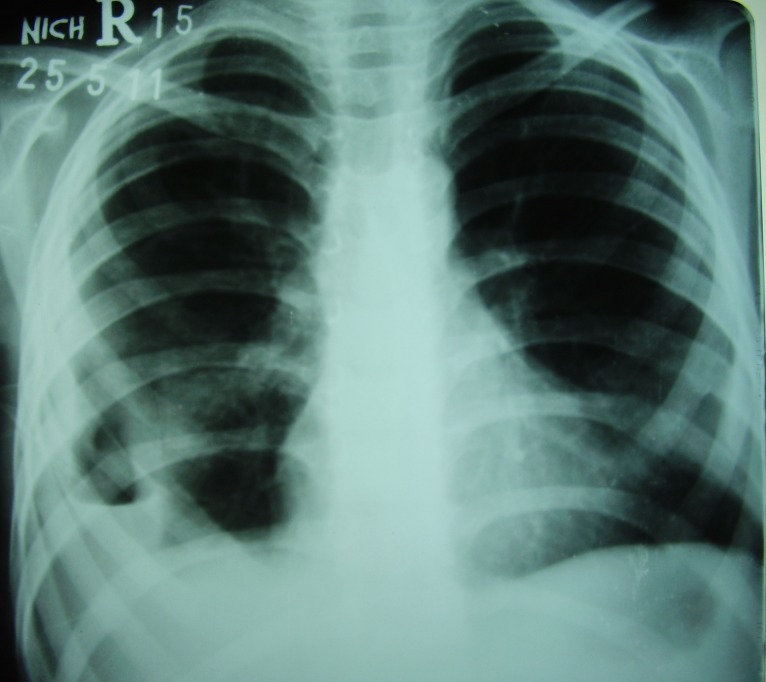
Figure 4: X-ray chest satisfactory lung expansion.

## DISCUSSION

Empyema thoracis continues to be a serious health problem especially in developing countries like ours, where health seeking behavior is poor and late referral to tertiary care centers is common. Inappropriately treated ET is associated with a mortality rate of 10 – 16 % [4]. Bilateral ET is infrequently reported in children. In a comparative review of 243 children with ET, Baranwal et al found a frequency of 5% of bilateral empyemas [5]. In another study, Bhatta et al reported 7.7% BET among 39 children with empyema thoracis [6], while none of the 79 patients with ET managed by Gün et al had bilateral disease [1]. The bilateral involvement suggests tuberculosis or parasitic infection according to the British Thoracic Society (BTS) guidelines for the management of pleural infection in children [7]. The symptomatology, morbidity and mortality increases with bilateral empyemas so was in our patient who had a stormy course and a prolonged hospital stay before improvement was seen.

In areas with high incidence, tuberculosis is one of the common causes of pleural effusion in children. It is characterized by exudative effusion more commonly involving right side, rarely bilateral. More often than not, there is history of close contact with tuberculosis and absent BCG vaccination history and scar. Our patient presented with an acute illness and classical symptoms (high grade fever, cough, dyspnea, anorexia and malaise) linked with tuberculous ET [3]. Moreover, she had history of contact with TB, was unvaccinated, had bilateral exudative effusions, all reminiscent of tuberculosis, as per BTS guidelines [7]. No other clue to tuberculosis could be found except lymphocytosis in initial pleural fluid RE and raised ESR. 

The optimal treatment of ET in children is debatable. It varies from antibiotics, thoracocentesis, tube thoracostomy, intra-pleural fibrinolytic agents, and open window thoracostomy to decortications. Results of management with tube thoracostomies and antibiotics vary among different studies. Some have recommended it for any stage of empyema while others advocate early intervention [7,8]. We opted for conservative management with bilateral tube thoracostomies, along with antituberculous drugs and other antibiotics according to pus culture reports of pleural fluid. Patient showed clinical and radiological improvement with this approach. Prolonged tube thoracostomies increase the overall morbidity, duration of hospital stay, cost of in-patient treatment and risk of nosocomial infections. Similar situation was reported by Brohi et al in one of their patients, who was continued with chest tube for 60 days [9].

## Footnotes

**Source of Support:** Nil

**Conflict of Interest:** None declared
